# Trabectedin arrests a doxorubicin-resistant PDGFRA-activated liposarcoma patient-derived orthotopic xenograft (PDOX) nude mouse model

**DOI:** 10.1186/s12885-018-4703-0

**Published:** 2018-08-20

**Authors:** Tasuku Kiyuna, Yasunori Tome, Takashi Murakami, Kei Kawaguchi, Kentaro Igarashi, Kentaro Miyake, Masuyo Miyake, Yunfeng Li, Scott D. Nelson, Sarah M. Dry, Arun S. Singh, Tara A. Russell, Irmina Elliott, Shree Ram Singh, Fuminori Kanaya, Fritz C. Eilber, Robert M. Hoffman

**Affiliations:** 1 AntiCancer Inc., San Diego, CA USA; 2 Department of Surgery, University of California, San Diego, CA USA; 3 Department of Orthopedic Surgery, Graduate School of Medicine, University of the Ryukyus, Okinawa, Japan; 4 Division of Hematology-Oncology, University of California, Los Angeles, CA USA; 5 Department of Surgery, University of California, Los Angeles, CA USA; 6 Division of Surgical Oncology, University of California, Los Angeles, CA USA; 7 Basic Research Laboratory, National Cancer Institute, Frederick, MD USA

**Keywords:** Liposarcoma, Patient-derived orthotopic xenograft, PDOX, PDGFRA amplification, Trabectedin, Precision medicine

## Abstract

**Background:**

Pleomorphic liposarcoma (PLPS) is a rare, heterogeneous and an aggressive variant of liposarcoma. Therefore, individualized therapy is urgently needed. Our recent reports suggest that trabectedin (TRAB) is effective against several patient-derived orthotopic xenograft (PDOX) mouse models. Here, we compared the efficacy of first-line therapy, doxorubicin (DOX), and TRAB in a platelet-derived growth factor receptor-α (PDGFRA)-amplified PLPS.

**Methods:**

We used a fresh sample of PLPS tumor derived from a 68-year-old male patient diagnosed with a recurrent PLPS. Subcutaneous implantation of tumor tissue was performed in a nude mouse. After three weeks of implantation, tumor tissues were isolated and cut into small pieces. To match the patient a PDGFRA-amplified PLPS PDOX was created in the biceps femoris of nude mice. Mice were randomized into three groups: Group 1 (G1), control (untreated); Group 2 (G2), DOX-treated; Group 3 (G3), TRAB-treated. Measurement was done twice a week for tumor width, length, and mouse body weight.

**Results:**

The PLPS PDOX showed resistance towards DOX. However, TRAB could arrest the PLPS (*p* < 0.05 compared to control; *p* < 0.05 compared to DOX) without any significant changes in body-weight.

**Conclusions:**

The data presented here suggest that for the individual patient the PLPS PDOX model could specifically distinguish both effective and ineffective drugs. This is especially crucial for PLPS because effective first-line therapy is harder to establish if it is not individualized.

## Background

Pleomorphic liposarcoma (PLPS), a type of sarcoma, is a rare and an aggressive variant of liposarcoma. PLPS is a recalcitrant disease. Patients with PLPS develop an elevated level of local recurrence and distant metastasis with poor prognosis [1]. PLPS consists of approximately 10% of liposarcomas. PLPS has been investigated in soft tissue sarcomas (STS) such as head and neck sarcoma as well as bone sarcoma [2, 3] and has been demonstrated in patients of all ages [4–7]. Wang et al. [8] reported 6 PLPS cases out of a total 89 liposarcoma cases between 2003 and 2017. All 6 patients underwent complete tumor resection and only one patient received chemotherapy with ifosfamide and epirubicin [8]. A primary PLPS in an 18-year-old male in the metaphysis of the left tibia was reported by Tiemeier et al. [3]. The patient was treated with methotrexate, doxorubicin and cisplatinum (MAP). Pathology results demonstrated extensive (> 95%) tumor necrosis due to neoadjuvant chemotherapy. Chemotherapy and post-operative results after 12 months showed no sign of recurrence [3]. Yan et al. [9] have shown an 81-year-old Chinese woman with advanced PLPS who was treated with apatinib after failure of chemotherapy had good efficacy and low toxicity. Apatinib was also used in patients with advanced sarcoma [10]. In search of molecular biomarkers, Ghadimi et al. [11] tested 155 PLPS patients using tissue microarrays, and identified several potential therapeutic targets [11].

Surgical resection at present is the only effective therapeutic option for localized PLPS. In addition, radiation and chemotherapy are largely ineffective for advanced stages of this disease [11–14]. Thus, for patients, personalised and targeted therapy is necessary to overcome the metastatic PLPS.

To accomplish this goal, our laboratory has developed the patient-derived orthotopic xenograft (PDOX) nude- mouse model for many tumor types [15–49]. Our PDOX model is advantageous compared to subcutaneous (s.c.)-transplanted patient-derived xenograft (PDX) models in various aspects [50, 51]. In contrast to PDOX model, s.c.-transplanted PDX models fail to develop an advanced tumor stage and cannot retain the original disease pattern generally observed in patients, PDOX models metastasize because tumor tissues are engrafted in the orthotopic sites of origin [50–52]. Importantly, the metastasic form of PDOX model matches the patient. Even though high technical skill, time commitment, and more costly procedures are needed for the PDOX models compared to traditional subcutaneous PDX models, PDOX models are important for individual patients and can be used as a powerful tool in preclinical modelling [50–52].

Trabectedin (TRAB) for patients with metastatic liposarcoma has been approved by the FDA [53] and is marketed by Janssen Pharma as Yondelis [54]. TRAB is a tetrahydroisoquinoline alkaloid compound, derived from the Carribean sea tunicate, *Ecteinascidia turbinate* [7, 55]. TRAB is a promising antitumor agent [56–59]. Nteli et al. [60] reported a durable response to TRAB in a patient with high-grade uterine leiomyosarcoma. It was reported that cells lacking a homologous recombination system were more sensitive to TRAB [61]. Subsequently, Larsen et al. [62] found that an interaction between a minor groove (DNA), and transcription factors, or DNA-repair molecules such as BRCA1, with TRAB alters the cell cycle and induces cell death. TRAB also showed anti-inflammatory and immunomodulatory properties [62]. Several studies reported that a haplotype in the *BRCA1* gene could be utilized as a marker for predicting TRAB effectiveness in patients with STS [63–65]. Angarita et al. [66] reported some efficacy of TRAB to advanced STS patients who did not respond to first-line chemotherapy.

Recently, we showed that TRAB is efficacious on several PDOX models [25, 30, 32, 67]. Here, we tested the efficacy of first-line chemotherapy, doxorubicin (DOX) [42], and TRAB in a PDGFRA-amplified [68] PLPS PDOX model [69].

## Methods

### Mice

In the present study, athymic *nu/nu* nude mice, between 4 and 6 weeks old, were utilised [69]. Experimental procedures and data collection were done as per as our previous publications [22, 24, 30, 32, 38, 47, 69, 70]. Mouse housing, feeding, surgical processes and imaging were conducted as described in our previous publications [22, 24, 30, 32, 38, 47, 69, 70]. The mice were humanely sacrificed as described in our previous publications [22, 24, 30, 32, 38, 47, 69, 70]. The mouse investigations presented here were done using an AntiCancer, Inc. Institutional Animal Care and Use Committee (IACUC)-protocol specifically approved for this study as previously described [47] and as per as principles and procedures provided in the National Institute of Health (NIH) Guide for the Care and Use of Animals under Assurance Number A3873–1 [47].

### Patient-derived PLPS tumor

In this study, we used a PLPS tumor derived from a 68-year-old male patient diagnosed with a recurrent PLPS, which has been described in our previous publication [69]. Details of surgical resection and chemotherapy given to this patient have been previously described [69].

### Establishing the PLPS PDOX model using the surgical orthotopic implantation (SOI) technique

PLPS sample collection from the patient, performing subcutaneous implantation in nude mice, harvesting tumors from the mice, creating a space at the orthotopic site in the biceps femoris to insert tumor fragments in the mice and to establish the PDOX model and wound-closure procedures have been described in detail in our previous publications [22, 24, 30, 32, 38, 47, 69].

### Treatment regime

All treatment procedures and data collection were done as previously reported [22, 24, 30, 32, 38, 47]. PLPS PDOX mouse models were randomized into three groups as previously described [69]: Group 1 (G1), control (untreated); Group 2 (G2), DOX-treated (3 mg/kg, i.v., weekly for 2 weeks); Group 3 (G3), TRAB-treated (0.15 mg/kg, i.v., weekly for 2 weeks). In each group, 7 mice were used. Measurement of tumor width, length, and mouse body weight was done as described in our previous publications [22, 24, 30, 32, 38, 47, 69]. The doses and treatment time were selected from our previous PDOX studies [22, 24, 30, 32, 38, 47, 69]. DOX was selected because it is first-line therapy for PLPS.

A formula to calculate the tumor volume has been previously described [69]. All data are presented in the results section as mean ± SD. Drug treatment was started only when the tumor volume attained 50 mm^3^ [69]. The tumor volume ratio and the actual body weight was measured as defined in our previous publications [22, 24, 30, 32, 38, 47, 69]. Mice were sacrificed on day 15 in each drug-treatment group and tumors were resected for further histological analysis as described in our previous publication [69].

### Histopathological evaluation

All histological procedures, data collection, and analysis were done as previously reported [22, 24, 30, 32, 38, 47, 71].

### Statistical analysis

All statistical analyses were done using JMP pro version 12 [69]. The relative tumor volumes and relative body weight of the mice are presented as mean ± SD [69]. The Mann-Whitney U test was used to confirm the significant differences for continuous variables. *P* values of less than 0.05 were regarded as statistically significant.

## Results

### Drug efficacy in the PLPS PDOX mouse model

**Fig. 1:**
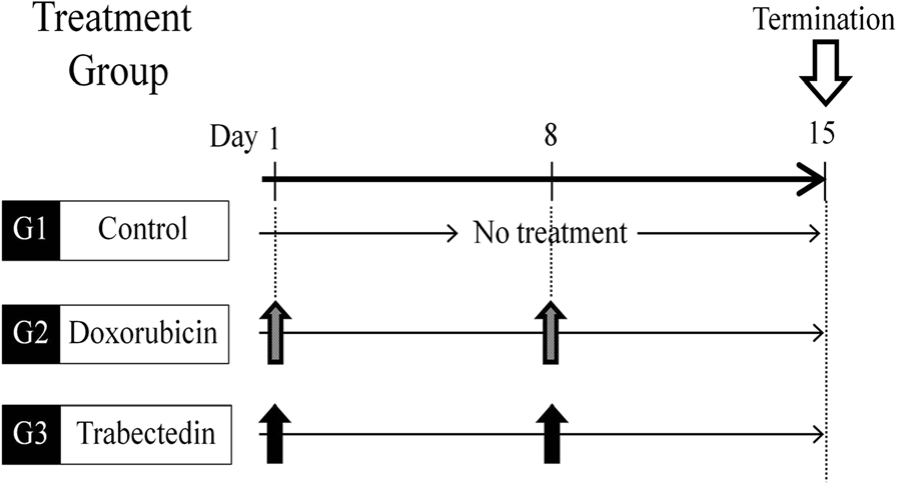
Treatment protocol and quantitative drug efficacy. Treatment protocol. G1: untreated control (*n* = 7); G2: treated with doxorubicin (DOX) (3 mg/kg, i.v., weekly, 2 weeks, *n* = 7); Group 3, treated with trabectedin (TRAB) (0.15 mg/kg, i.v., weekly, 2 weeks, *n* = 7). All treated mice were sacrificed on termination day-15, and tumors were resected for further histological evaluation

**Fig. 2:**
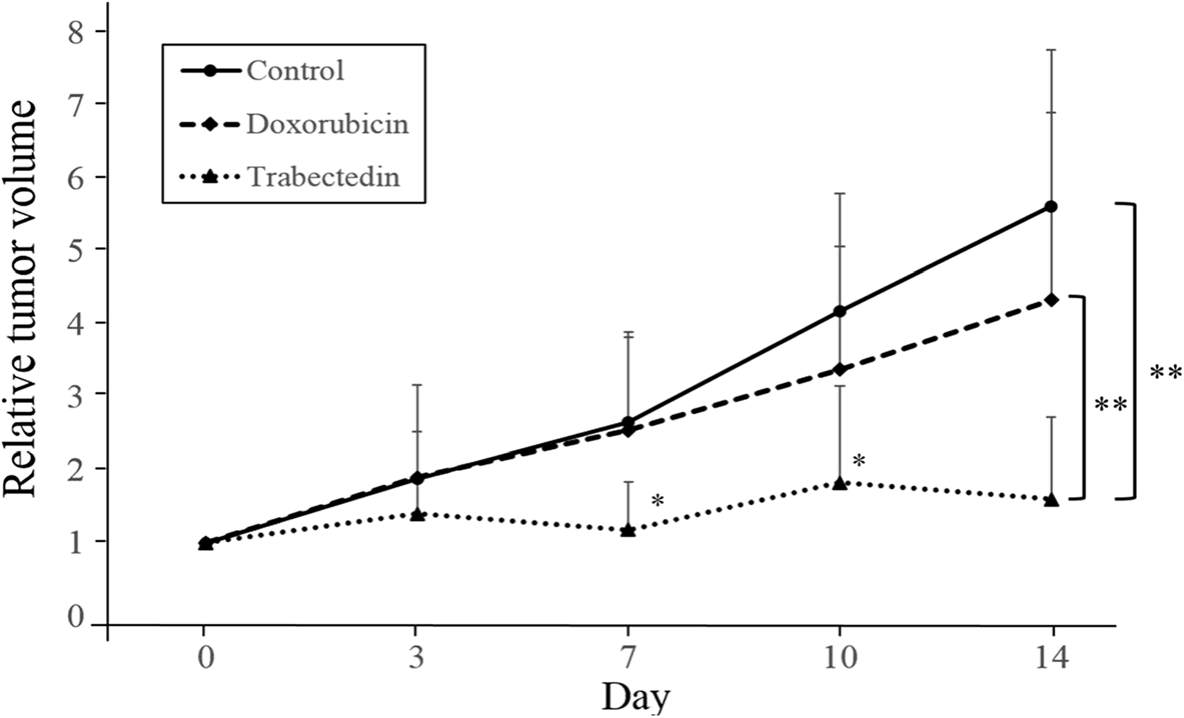
Line graphs show relative tumor volume (tumor at any time point relative to day 0). *p < 0.05, **p < 0.01, Error bars: ±SD

**Fig. 3:**
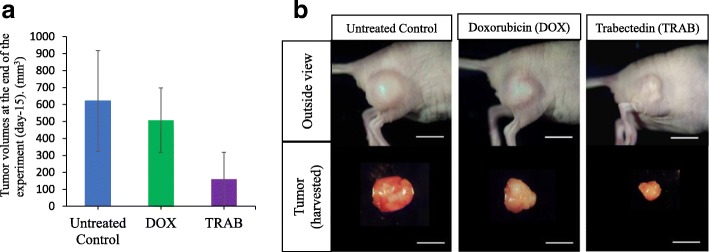
**a** Tumor volumes at the end of the experiment (day-15). **b**: Macro tumor images after treatment. All treated mice were sacrificed on day-15, and tumors were resected for further histological evaluation. Images are representative of tumors harvested after orthotopic growth in the biceps femoris. Scale bar: 10 mm

To test the efficacy of each drug in the PLPS PDOX mouse model, two weeks following orthotopic implantation, mice with tumors were randomized into three groups to initiate treatment (Fig. 1). We found that in the control group (G1-untreated) tumors grew more than five times larger by day 14 compared to day 0 (tumor-volume ratio = 5.61 ± 2.14). In the DOX-treated group (G2), on day 14, we could not observe a significant reduction of tumor growth [69] compared to the control group (tumor-volume ratio = 4.33 ± 2.57, *p* = 0.927). In contrast, TRAB (G3) treatment showed significant tumor-growth inhibition on day 14 (tumor-volume ratio = 1.60 ± 1.13, *p* = 0.0032 compared to the control). In addition, on day 14, TRAB treatment also resulted in more suppression of tumor growth than DOX treatment (*p* = 0.0092) (Fig. 2). It took two weeks for the tumor to initially grow to 50 mm^3^. The tumor volumes at the end of the experiment were: untreated control, 621 ± 297 mm^3^; DOX, 507 ± 191 mm^3^; TRAB, 159 ± 95 mm^3^ (Fig. 3a).

Figure 3b shows representative images of tumors harvested from the biceps femoris at the termination point on day 15. The control group had the largest tumors and the TRAB groups had the smallest tumors (Fig. 3b).

### Effect of drug treatment on body weight

**Fig. 4:**
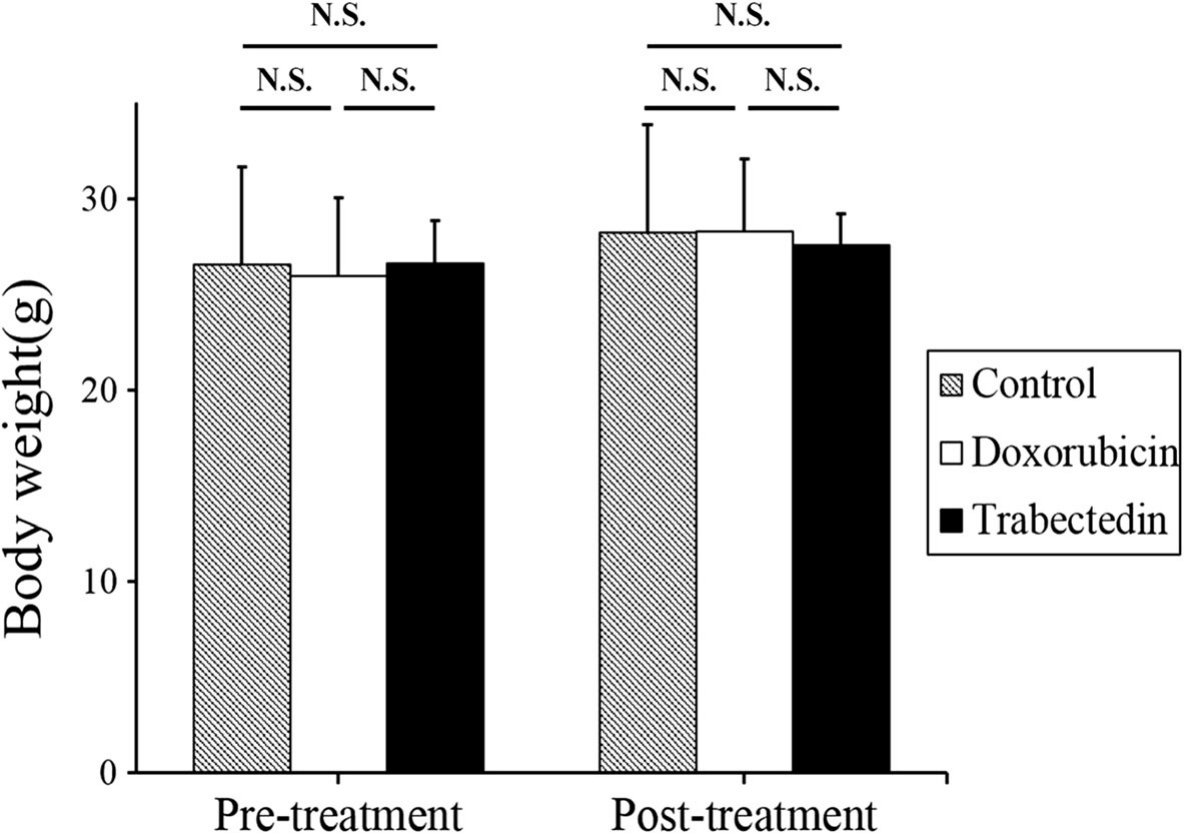
Mouse body weight. Bar graphs show body weight of mice treated with each compound as well as the untreated control. Error bars: ± SD. n.s.: not significant

We measured the mouse body weight pre-treatment (before) as well as post-treatment (after). We did not find any significant differences in the body weight between any treatment group (Fig. 4).

### Effect of drug treatment on tumor histology

**Fig. 5:**
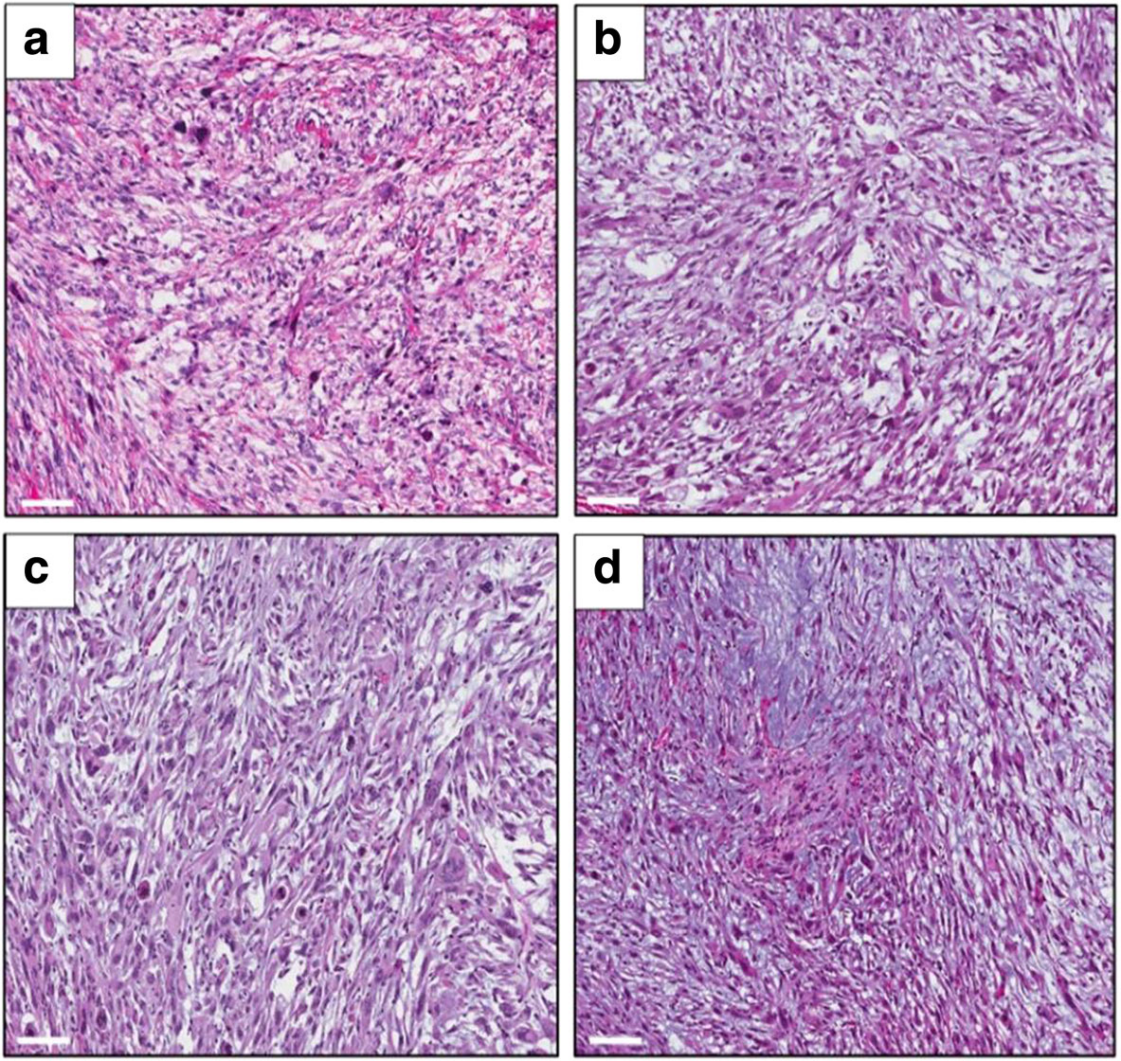
Tumor histology. Hematoxylin and eosin (H&E), a stained sections of tumors. **a**: original patient tumor; **b**: untreated control PDOX tumor; **c**: DOX-treated PDOX tumor; **d**: TRAB- treated PDOX tumor. Scale bars: 100 μm

We analysed the tumor histology in the untreated (control) and treated tumors (treated with DOX or TRAB). Photomicroscopy results showed that both the original patient tumor and untreated control-group tumor had enlarged and hyperchromatic nuclei with cytoplasmic vacuoles that are usual in PLPS [69] (Fig. 5a and b). Further, the untreated (control) PDOX tumor exhibits normal and viable cancer cells in nearly all areas [69] (Fig. 4b). In contrast to the DOX-treated tumor that did not show necrotic areas (Fig. 5c), the TRAB-treated tumor (Fig. 5d) shows extensive necrosis (Fig. 5c).

## Discussion

First-line systemic therapy for PLPS with an anthracycline, such as a DOX-containing regimen, has a low response rate [72] consistent with our results presented here. We found that the PLPS PDOX showed resistance to first-line therapy DOX, which is similar to our previous study [69]. In contrast, the PLPS PDOX was arrested by TRAB. This suggests that PLPS PDOX model could specifically recognize both effective and ineffective drugs for each patient [69].

TRAB is a novel marine-derived alkaloid [7, 73]. It attaches covalently to the DNA minor groove and interacts with transcription factors [74]. Recently, Pignochino et al. [75] demonstrated TRAB and Poly [ADP-ribose] polymerase 1 (PARP1 1) inhibition synergism in sarcomas. Laroche et al. [76] tested the efficacy of a combination of TRAB and rucaparib on STS and found that they were also synergistic, enhanced apoptosis and blocked the cell cycle. In addition, these combinations were more effective than TRAB and rucaparib alone [76]. Further, they observed that the combination of these two drugs resulted in elevated γH2AX intranuclear accumulation, which is due to DNA-damage induction [76]. In vivo results further demonstrated that combining these two drugs significantly improved disease-free survival with massive tumor necrosis [77]. A few studies also showed that TRAB has anti-tumor and anti-inflammatory activities [77, 78] and can selectively lower monocytes, tumor-associated macrophages (TAM), and angiogenesis [77]. It has also been demonstrated that the therapeutic efficacy of TRAB in osteosarcoma is increased in combination with a PD-1-blocking antibody [79].

TRAB has been demonstrated as a therapeutic option in STS [56, 57, 80–86], recurrent ovarian cancer [87], metastatic breast cancer [88], solitary fibrous tumor (SFT)-PDXs [89], desmoplastic small round cell tumor [90], juvenile myelomonocytic leukaemia and chronic myelomonocytic leukaemia [91]. Recently, we reported that TRAB is efficacious on an osteosarcoma cisplatinum-resistant lung metastasis [67], a BRAF-V600E mutated melanoma [30, 32] and a gemcitabine (GEM)-resistant pancreatic cancer [25].

Here we showed that TRAB was highly effective against a PDOX model of PLPS with a PDGFRA activating mutation [68] in comparison to DOX. The above results together suggest the improved clinical prospect of PLPS and the importance of individualized therapy with PDOX models [70]. Experiments will be performed in the future to compare TRAB with other first-line therapies of PLPS such as docetaxel and ifosfamide. Whether TRAB will be effective against unresetable PLPS is an experimental question since drug sensitivity is highly specific for each patient. It will be possible to answer this question in the future with a PDOX model derived from a core-needle biopsy.

## Conclusions

Our results suggest that the PLPS PDOX model [69] can precisely distinguish both efficacious and non-efficacious drugs for each patient. These results are crucial for PLPS, which is a heterogeneous group, where effective first-line therapy is difficult to establish if it is not individualized [69].
